# Clinical Characteristics and Outcomes of Tympanomastoid Paragangliomas: A Report from Slovenia

**DOI:** 10.3390/cancers16183178

**Published:** 2024-09-17

**Authors:** Manja Hribar, Iztok Fošnarič, Aleš Matos, Robert Šifrer, Aleš Grošelj, Maruša Debeljak, Nina Zidar, Primož Strojan, Klemen Jenko

**Affiliations:** 1 Department of Otorhinolaryngology and Cervicofacial Surgery, University Medical Centre Ljubljana, Zaloška cesta 2, 1000 Ljubljana, Slovenia; manja.hribar@kclj.si (M.H.);; 2 Faculty of Medicine, University of Ljubljana, Vrazov trg 2, 1000 Ljubljana, Slovenia; 3 Clinical Institute for Special Laboratory Diagnostics, University Children’s Hospital, University Medical Centre Ljubljana, Bohoričeva ulica 20, 1000 Ljubljana, Slovenia; 4 Institute of Pathology, Faculty of Medicine, University of Ljubljana, Korytkova 2, 1000 Ljubljana, Slovenia; 5 Department of Radiotherapy, Institute of Oncology Ljubljana, Zaloška cesta 2, 1000 Ljubljana, Slovenia

**Keywords:** paraganglioma, jugulotympanic, tympanic, treatment

## Abstract

**Simple Summary:**

Temporal bone paragangliomas are rare neuroendocrine tumors that typically arise from the parasympathetic nervous system. Since they are not a common pathology in the daily practice of an otorhinolaryngologist, we would like to provide an overview of current treatment strategies for patients with tympanomastoid paragangliomas. In addition, we present our approach for patients with class A and B tympanomastoid paragangliomas and reiterate that surgical therapy is an effective and safe treatment modality. In cases where surgery is refused and the tumor has grown to class C or more, radiotherapy is an alternative method to control tumor growth.

**Abstract:**

(1) Background: Head and neck paragangliomas are neuroendocrine tumors that typically originate from the parasympathetic nervous system and are predominantly non-secretory. Their clinical manifestations result from their mass effect on the surrounding tissues. The approach to treating these tumors depends on factors such as their location, size, impact on adjacent structures, and the patient’s overall health and preferences. (2) Methods: A retrospective analysis of the management of temporal bone paraganglioma classes A and B (according to the modified Fisch classification) was performed at the University Medical Centre, Ljubljana, between 2011 and 2023. (3) Results: We analyzed 23 cases, 19 of which underwent surgery; complete tumor removal was achieved in 18 of them. Four patients were irradiated due to tumor progression to class C. Three of these four patients initially refused surgery and were treated with radiotherapy (RT) 7, 13, and 18 years after diagnosis. In the fourth patient, complete surgical resection was not achieved and she was treated with RT four years after surgery, due to the growth of the tumor to class C. The average follow-up time from diagnosis was 8.9 years (median 6 years; range 1–26 years). (4) Conclusions: The surgical treatment of patients with class A and B paragangliomas is effective and safe. In cases where surgery is refused but the tumor continues to grow to class C, RT is an alternative and efficient method of controlling tumor growth.

## 1. Introduction

Paragangliomas (PGLs) are neuroendocrine tumors that originate from the parasympathetic or sympathetic nervous system [1]. Their annual incidence is between 2 and 8 per million, with an estimated prevalence between 1:2500 and 1:6500 [2,3], peaking between the ages of 30 and 50 [4]. Approximately 85% of sympathetic PGLs manifest in the abdomen, predominantly in the adrenal gland as pheochromocytomas. In contrast, the majority of parasympathetic PGLs are localized in the head and neck area and are rarely hormonally active (1–3%) [5]. The symptoms of PGLs in the head and neck are primarily due to the mass effects on the surrounding tissue, as they typically do not secrete catecholamines.

**Figure 1:**
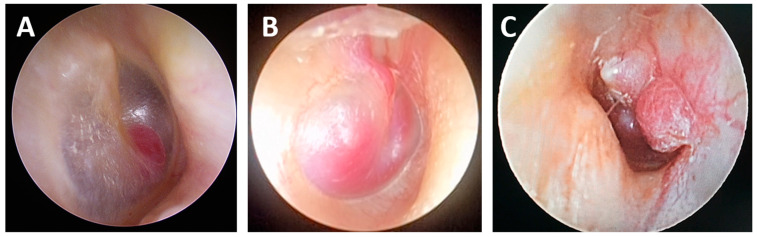
Endoscopic view of paraganglioma (PGL) class A1 (**A**), class B1 (**B**), and class B2 (**C**) behind the intact tympanic membrane.

PGLs originating from the temporal bone, including the tympanic, tympanomastoid, jugular, and jugulotympanic PGLs, may originate from the Jacobson’s nerve, Arnold’s nerve, or the adventitia of the jugular bulb. They typically manifest as a reddish, pulsatile mass behind the eardrum (Figure 1). It is important to distinguish them from similar changes, such as a high jugular bulb or an aberrant carotid artery in the middle ear. These tumors commonly present with pulsatile tinnitus, often synchronized with the heartbeat, as well as progressive hearing loss, dizziness, pressure or pain in the ear, and bleeding through the external auditory canal. As the tumor enlarges, adjacent structures such as the jugular bulb, internal carotid artery, facial nerve, and lower cranial nerves (IX, X, XI, and XII) may be damaged, resulting in dysphagia, hoarseness, limited tongue movement, and fasciculations and weakness when lifting the ipsilateral shoulder. In addition, advanced tumors can spread intracranially [6,7,8].

About half of the diagnosed head and neck PGLs show growth after diagnosis. The growth rate is typically slow and is estimated at 0.8 mm/year. PGLs of the temporal bone show the slowest growth, averaging 0.4 mm/year. However, the average growth rate of carotid and vagal PGLs is 1.6 mm/year [9,10].

At least 40% of PGLs are related to mutations in various genes, and genetic testing is recommended for all patients with PGLs [4,6,7,11]. Mutations in the subunits of the succinate dehydrogenase (SDH) gene (A, B, C, D, or AF2) are most common and determine the aggressiveness of the tumor. Tumors with *SDHB* mutations are the most aggressive, being malignant or metastatic in 23% of cases. They are associated with a higher risk of other primary tumors, especially renal cell carcinoma, papillary thyroid carcinoma, neuroblastoma and gastrointestinal stromal tumors [4,6,11], whereas individuals with an *SDHD* mutation are more likely to develop multiple PGLs throughout their lifetime, requiring individual management strategies to preserve lower cranial nerve functions [7,12].

To evaluate the primary lesion, patients diagnosed with PGL of the temporal bone should undergo magnetic resonance imaging (MRI) and high-resolution computed tomography (HRCT), which generally provide a better delineation of tumors located within the jugular foramen and hypotympanum than, e.g., magnetic resonance angiography (MRA) [4,10,11,13]. It is also recommended that all patients undergo diagnostic imaging of the chest, abdomen, and pelvis to look for metastases or secondary tumors, ideally by MRI or alternatively by gallium DOTATATE PET CT [4]. The most suitable whole-body imaging method for the primary detection of PGLs and for follow-up is still controversial [14].

The treatment of choice for tympanic and tympanomastoid PGLs is surgical removal [4,15]. Class A tympanic PGLs can usually be removed via the ear canal, whereas class B PGLs typically require an approach through the mastoid [16]. Preoperative embolization is indicated from class C temporal bone PGLs onward [4]. For class C temporal bone PGLs and larger tumors, the main indications for treatment include tumor growth and symptom control. Primary RT is only indicated for larger tumors that are difficult to remove surgically without damaging surrounding structures. There is currently no clear consensus on the preferred primary therapy, as surgery and RT are expected to provide equivalent long-term growth control [4].

Since temporal bone PGLs are an uncommon pathology, we conducted a retrospective analysis of patients with class A and B tympanomastoid PGLs treated at our institution, aiming to review their clinical characteristics, management, and outcome.

## 2. Materials and Methods

All patients with class A and B tympanomastoid PGLs (according to modified Fisch and Mattox classification by Sanna [17,18]) treated at the Department of Otorhinolaryngology and Cervicofacial Surgery of the Medical Centre, Ljubljana, Slovenia, between 2011 and 2023 were reviewed. Using the patient database, we identified all individuals with the terms ‘paraganglioma’ or ‘glomus’ recorded in their medical records. Some of the included patients underwent surgery before 2011, yet they were included in the study because, during the period covered, they attended follow-up appointments and/or required additional treatment. The medical records of identified patients were reviewed to collect information on the characteristics of patients and tumors, therapy, and disease outcomes.

Immunohistochemistry was performed according to standard methodology. We used commercially available antibodies against synaptophysin (clone MRQ-40, Cell Marque, Rocklin, CA, USA), chromogranin (clone Polyclonal, Dako, Glostrup, Denmark), cytokeratin (clone AE1/AE, Merck, Darmstadt, Germany), S100 (clone S10A1, Abcam, Cambridge, UK), Ki67 (clone MIB-1, Dako, Glostrup, Denmark), and SDHB (clone 21A11AE7, Abcam, Cambridge, UK). Staining was performed in the BenchMark XT immunostainer (Ventana Medical Systems, Tuscon, AZ, USA). After antigen retrieval with Cell Conditioning 1 buffer (Ventana Medical Systems, Tuscon, AZ, USA) at 95 °C for 30 min, the slides were incubated with primary antibodies against the antigens. The immunoreactivity was visualized using the iVIEW DAB Detection Kit (Ventana Medical Systems, Tuscon, AZ, USA), according to the manufacturer’s instructions. Sections were counterstained with hematoxylin. Positive controls, as suggested by the manufacturers, and negative controls omitting the primary antibodies were also included.

Genetic analysis was performed at the University Children’s Hospital, Ljubljana, utilizing whole exome sequencing (WES). Genomic DNA was isolated from peripheral blood according to the established protocols, using the FlexiGene DNA Kit 250 (Qiagen, Hilden, Germany). Libraries were prepared with the Illumina DNA Prep with Enrichment kit (Illumina, San Diego CA, USA) and xGen Exome Research Panel v2 probes (IDT, San Diego CA, USA). Next-generation sequencing (NGS) was performed using the NovaSeq S4 6000 Reagent Kit (Illumina, San Diego, CA, United States) according to the manufacturer’s instructions, and was sequenced on the NovaSeq 6000 sequencer (Illumina, San Diego, CA, United States). A panel of genes associated with PGL (panel app v1.11: *FH, MAX, MEN1, NF1, PRKAR1A, RET, SDHA, SDHAF2, SDHB, SDHC, SDHD, TMEM127, VHL, CDC73, CDKN1B, GDNF, KIF1B, MDH2, PTEN,* and *TP53*) was used for filtering variants. The Variant Analysis and Filtration Tool (VarAFT v.2.17-1) (Aix Marseille University, France) was utilized for annotation and filtration. *SDHAF2, SDHB, SDHC,* and *SDHD* genes were 100% covered for at least 20X; no copy number variations (CNVs) were detected.

## 3. Results

A total of 23 patients were identified who were ultimately diagnosed with PGLs class A or B, comprising 4 (17%) males and 19 (83%) females. In total, 13 (57%) patients had a PGLon the right side, while 10 (43%) had a tumor on the left side. None of the patients had bilateral tumors or PGL elsewhere in the ENT region. The average age of the patients at the time of diagnosis was 58 years (range: 39–85). The average follow-up time from diagnosis was 8.9 years (median 6 years; range 1–26 years) and the majority (65%) of the patients were followed up at a time point beyond 5 years.

### 3.1. Diagnostic Evaluation

In total, 22 out of 23 (96%) patients underwent imaging diagnostics (CT, MRI, MRA, or computed tomography angiography (CTA)) of the primary lesion. A total of 13 (57%) patients underwent both temporal bone CT and MRI, while 6 (26%) patients underwent only CT and 3 (13%) underwent only MRI of the primary lesion. Additionally, 9 (39%) patients also had angiography, with 4 (17%) undergoing MRA and 5 (22%) undergoing CTA.

In one patient, imaging diagnostics were not performed before the surgery. This patient had mastoid exploration due to a polyp in the ear canal, which was later diagnosed as PGL. Preoperative embolization was performed in 6 patients; in 2 of these patients, this was only performed during the second surgery due to tumor regrowth.

According to the expanded Fisch classification [18], four patients had A1 tumors, five A2, nine B1, and three B2 at the time of diagnosis. In 2 patients, the initial stage could not be determined; however, they had B2 tumors at the time of the recurrence (surgery was carried out 2 and 20 years before). For the ease of presentation of the results, we counted them as B2. Laboratory tests did not confirm secretory activity among our patients.

### 3.2. Clinical Presentation

**Figure 2:**
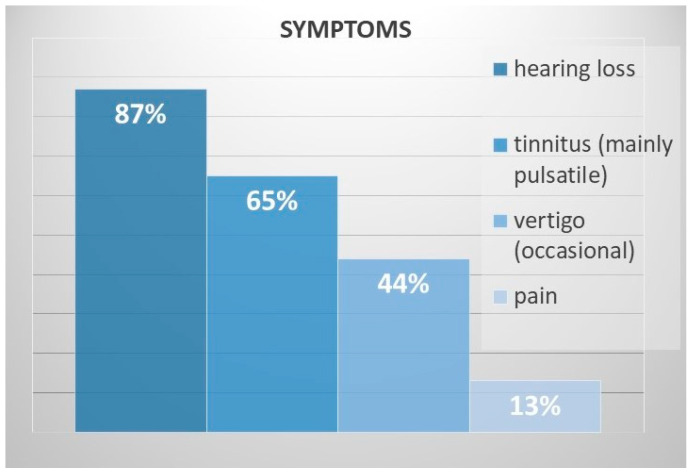
Symptoms of patients with class A and B PGLs.

The most common symptoms were hearing loss (20/23, 87%) and tinnitus (15/23, 65%), which was mostly pulsatile (Figure 2). Vertigo was occasionally experienced by 10/23 (44%) patients, and ear pain or discomfort was experienced by 3/23 (13%) patients. None of the patients presented with facial nerve or lower cranial nerve palsy.

### 3.3. Management and Treatment

**Figure 3:**
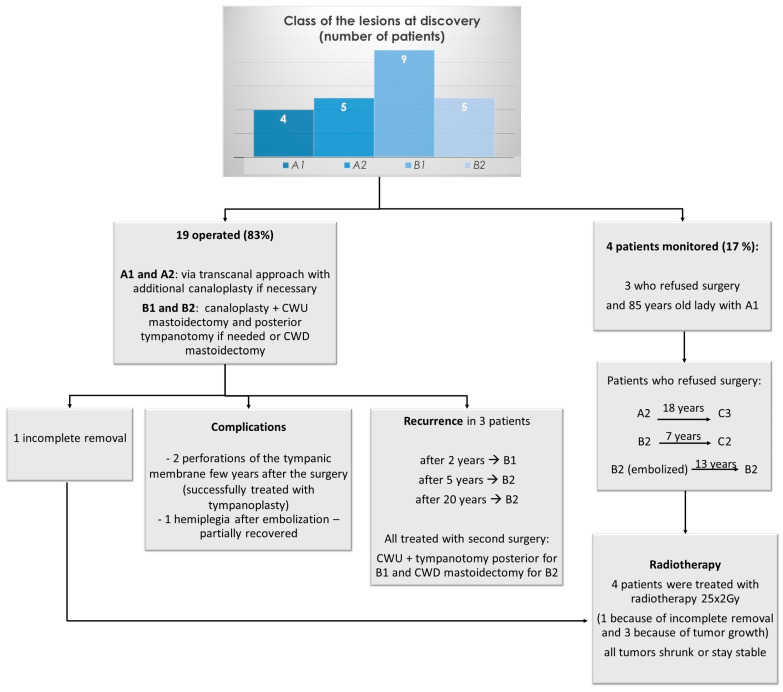
Management of patients with class A and B PGLs.

The distribution of tumors in classes according to the modified Fisch classification at diagnosis and their treatment is presented in Table 1 and Figure 3.

#### 3.3.1. Surgery

**Figure 4:**
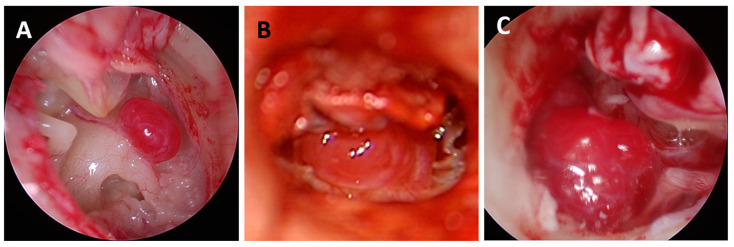
Intraoperative view of class A1 (**A**), class A2 (**B**), and class B1 (**C**) PGLs after canaloplasty and tympanomeatal flap elevation.

With the exception of one elderly patient, all patients with PGLs (class A or B) were offered or advised surgical intervention. Three of these patients declined surgical treatment and an 85-year-old woman with an A1 PGL opted for tumor monitoring due to the small size of the lesion, absence of symptoms, and age; after two years, no increase in size was observed. Nineteen patients were operated on. The approach to stage A tumors was transcanal for the majority; for stage B tumours, the approach was a combined transcanal with canal wall up mastoidectomy and posterior tympanotomy, if needed (Figure 4). The removal of the external auditory canal wall (canal wall down mastoidectomy) for better tumor visualization was necessary in three patients; two of these occurred during recurrence surgery. PGLs were removed mainly with bipolar electrocautery under the microscope, with the additional help of the endoscope if needed. There were no immediate postoperative complications. However, two patients later experienced tympanic membrane perforation, which was successfully treated with tympanoplasty. In one patient, ischemia occurred in the basal ganglia during embolization, resulting in hemiplegia, which only partially recovered; the function of the left arm was not fully rehabilitated, while rehabilitation of the left leg was successful.

Complete tumor removal was achieved in 18 out of 19 patients (95%). In one patient, the tumor was not completely removed at a site where it had invaded the cochlea. She underwent exploratory surgery for a polyp in the external auditory canal. At the time of surgery, canal wall down mastoidectomy was performed and the underlying pathology was not known to be PGL. Four years later, the patient was treated with radiotherapy (RT) due to tumor regrowth to a C2De1-class PGL.

In 3 patients, recurrence occurred 2, 5, and 20 years after surgery. In each case, the recurrent tumor grew to a size comparable to the original size before surgery (B1 or B2). All 3 patients underwent additional surgery, and no further recurrence was observed in any of them during follow-up (1, 4, and 6 years after the second operation).

#### 3.3.2. Radiotherapy

A total of 4 out of 23 patients (17%) received RT—three patients who refused surgery and the patient with incomplete tumor removal. The patient diagnosed with an A2 tumor and who was also being treated for schizophrenia refused regular follow-up appointments for 13 years. After that, annual MRI scans for the first 4 years showed no significant changes, but then, the tumor began to grow outward from the ear canal, exhibiting a visible 3 × 5 × 5 cm bleeding mass (class C3 tumor) at the time of RT. In the second patient who refused surgery with a class B2 PGL, tumor growth was detected during annual MRI monitoring 5 years after diagnosis, which expanded to a class C2 tumor within 2 more years and required RT. In the third patient with a B2 tumor, complications following tumor embolization were the reason for refusing surgery. She underwent RT 13 years after diagnosis of PGL due to the slow tumor growth. The fourth patient with an incompletely removed PGL was irradiated 4 years after the operation due to renewed tumor growth to C2De1.

All patients had CT-based RT planning with 5-point thermoplastic head masks for immobilization and were irradiated with a 6 MV photon beam from a linear accelerator (Varian Medical Systems, Inc, Palo Alto, CA, USA), using an intensity-modulated radiotherapy (IMRT) or volumetric-modulated arc therapy (VMAT) technique. For planning purposes, simulation contrast-enhanced CT scans (slice thickness of 2–3 mm) were fused with MRI scans, with T1 and T2 sequences (slice thickness of 1.7–2.9 mm) acquired within the next three days in the treatment position to better visualize the tumor and adjacent organs at risk. Only the tumor was irradiated with a gross tumor volume (GTV) to the planning target volume (PTV) margin of 5 mm. No clinical target volume (CTV) was created. The list of marked organs at risk, dosimetric constraints, dose prioritization, and acceptance criteria are shown in Table 2. A dose of 50 Gy was delivered in 25 fractions over 5 weeks (1 fraction/day, 5 days/week) to all patients. Only the patient with a bleeding tumor that was growing out of the ear canal first received a boost dose of 2 × 4 Gy with 15 MeV electron beams through a direct opposing field, which successfully stopped the bleeding. One week later, she began photon irradiation as described above.

During and after RT, one patient experienced grade 1 radiodermatitis according to the Common Terminology Criteria for Adverse Events of the National Cancer Institute, two patients experienced transient otorrhea, and one of them also experienced a transient sensation of first-degree pain in the ear. In a patient with a large tumor protruding from the auditory canal, a partial stenosis of the external auditory canal developed over the years with complete regression of the tumor outside the ear and in the external auditory canal. No facial and lower cranial nerve dysfunction was observed during follow-up, and in all patients, annual MRI scans confirmed that tumor growth was halted or even reduced in volume 4 to 6 years (median 4.5 years) after RT.

### 3.4. Histology and Genetics

**Figure 5:**
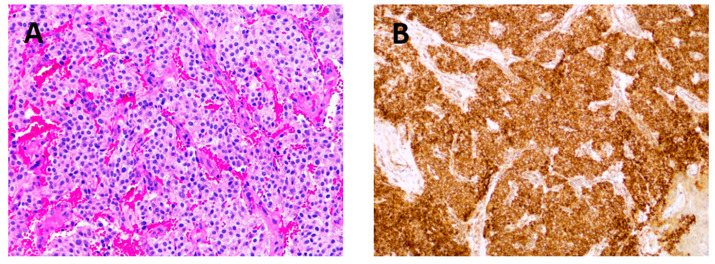
Histopathological images of PGL. Tumor is composed of islands of tumor cells with pale eosinophilic cytoplasm and slightly atypical nuclei, with highly vascular stroma (**A**). Immunohistochemical reaction for SDHB is positive in all tumor cells (**B**). Orig. magnification—10x.

All PGLs were histopathologically confirmed and showed a typical morphology and immunohistochemical profile (positive reaction for chromogranine and/or synaptophysin in tumor cells and for S100 in sustentacular cells; negative reaction for cytokeratin). In all samples, the immunohistochemical reaction for SDHB was positive (Figure 5) and the Ki67 proliferation index (mitotic index) was low. Twelve samples from this cohort were older than 8 years and were stained subsequently, as SDHB immunohistochemistry was not conducted routinely in all samples in the past. In these older samples, the staining for SDHB was occasionally weak, but was considered positive. Immunohistochemistry in old biopsy tissue samples may be unreliable as studies have shown that immunohistochemical intensity decreases significantly after 6–8 years of storage [19,20].

Genetic testing was conducted in 7 patients (29%). All the results were negative.

## 4. Discussion

The present report on 23 patients with tympanomastoid PGL diagnosed and treated in Slovenia over a period of 13 year confirms that surgery is the treatment of choice for class A and B tumors. For patients who refuse surgery or are not suitable for major surgery but with growing tumors and whose tumors progress to stage C, RT is a safe and effective treatment alternative.

In our cohort, the average age at tumor detection was 58 years, the male-to-female ratio was 1 to 4, and the most common symptoms were hearing loss, tinnitus, and vertigo, which is consistent with the published literature [15,21,22,23]. Good oncologic and functional results of primary surgery can be achieved with proper surgical technique and good exposure of the tumor [15,24]. Class A tympanic PGLs can usually be removed via the ear canal (with adequate canaloplasty if necessary), whereas class B PGLs typically require additional access through the mastoid. For easier access to the tumor and better control of the surrounding structures, a glove finger flap technique can be very useful [15]. Studies show that complete resection is possible in approximately 94–100% of cases. If the tumor is adherent to critical structures (facial nerve, cochlea, and carotid artery) and its removal would cause significant harm to the patient, it is more advisable to leave a portion of the tumor in place and monitor the patient [21,22,25]. In our patients, complete resection was possible in 19/20 patients (95%). Recurrence occurred in 3 (15%) patients postoperatively at 2, 5, and 20 years post-surgery. The recurrence rate of PGL reported in the literature varies between 0 and 15% for tympanic PGLs (17% for Fisch stages C and D) [15,22,26,27]. Recently, a study was published involving 173 patients with 189 PGLs in the head and neck region. The 10-year recurrence rate after therapy (surgical or radiation) was 28.2% for jugular PGLs, 12.4% for carotid PGLs, 10.5% for vagal PGLs, and 9.7% for tympanic PGLs [28]. The slightly higher recurrence rate in our case series is probably due to the smaller sample size and longer follow-up period compared to other studies [15,22,23].

There is no consensus on the follow-up monitoring of patients with completely resected PGLs. British guidelines suggest that radiologic surveillance can be discontinued after 5 years if no genetic mutations are present. However, due to the propensity for the recurrence of jugular tumors, continued surveillance beyond this period is recommended [4]. A study investigating recurrence rates and the growth of PGL recommended periodic imaging every few years and surveillance over several decades [28].

Tumor growth was observed in all our patients who refused surgery. This shows that it is advisable to treat smaller tympanomastoid tumors surgically as early as possible. The treatment of larger tumors is much more difficult and there is a higher risk of damage to the cranial nerves either due to the size of the tumor itself or as a result of the surgery. The main indications for the treatment of larger temporal bone and jugular PGLs are tumor growth, control of symptoms, and prevention of the onset of symptoms or cranial nerve paresis. Surgery and RT are expected to provide equivalent long-term growth control [4]. There is no consensus on the preferred primary therapy in such cases. Surgical treatment usually requires the displacement of the facial nerve, resulting in additional (or new) impairment of nerve function, which may improve to some degree over time. Due to the proximity or involvement of the lower cranial nerves (IX, X, XI, and XII), postoperative paresis of these nerves can be expected in 0–36% of cases [26,29]. On the other hand, RT can produce xerostomia, mucositis, dermatitis, and nausea. It can also lead to bone or brain necrosis or the exacerbation of the cranial nerve dysfunction if the dose exceeds a certain threshold, which is very unusual for the dose range used in PGLs. Considering that RT has less severe treatment-related side effects compared to surgery, some recommend primary RT treatment, especially fractionated RT. For benign lesions such as PGLs, a dose of 45–50 Gy is recommended, while stereotactic radiosurgery in a dose range of 12–14 Gy is suitable for smaller tumors [4,10]. The Italian Gruppo Otologico primarily recommends surgical therapy for patients under 65 years of age. For older patients or inoperable tumors, their policy is “wait and scan”, and only after proven growth can RT or partial resection of the tumor with adjuvant RT be chosen [29].

Four patients in our series were treated with RT after the tumor had grown. In all cases, the growth stopped or the tumor even shrank over the next 4 to 5 years after RT. Given the slow growth rate of PGLs, the question arises as to the optimal timing for RT, considering that tumors can remain stable for longer periods of time and show no visible growth. In our experience, RT is indicated for large, growing tumors that are either inoperable or at high risk for major surgical complications, as well as for patients who are not suitable for major surgery or for those who refused surgery. Histologically, PGLs are characterized by distinct arrangements of tumor cells known as ‘zellballen’, which are highly vascular stroma and are peripherally located sustentacular cells that stain positively for S100. Mitotic figures are usually rare. These tumors typically exhibit negative staining for keratin but demonstrate positive immunohistochemical reactivity to neuroendocrine markers such as chromogranin, synaptophysin, and INSM1. More importantly, an immunohistochemical reaction for SDHB facilitates the identification of tumors associated with mutations in *SDHx* genes [1]. The immunohistochemical reaction to SDHB was positive in all samples, suggesting that there is probably no underlying mutation in *SDHB* or *SDHD*. In particular, immunohistochemical staining with SDHB antibodies allows the samples to be divided into SDHB-positive and -negative groups. A negative immunohistochemical result may indicate the presence of an *SDHB* mutation, while tumors with *SDHD* mutations show weak or absent staining. In an immunohistochemical analysis of 220 pheochromocytomas and PGLs, 102 tumors were associated with *SDHx* mutations, and the staining protocol had a sensitivity of 100% and a specificity of 84%. Considering the significant costs associated with genetic testing and the consistently negative immunohistochemical findings in *SDH*-mutated tumors, it has been suggested that genetic testing should be performed selectively only in cases showing SDH-negative immunohistochemistry [30]. Subsequent studies have reconfirmed the reliability and efficacy of immunohistochemistry as a diagnostic tool to identify *SDHx* mutations in PGLs [31]. Given the recent literature describing an increasing percentage of genetic mutations in PGLs, our results are somewhat surprising. This may be due to the fact that our study only included smaller tympanomastoid tumors (classes A and B), which are less likely to have a genetic origin compared to carotid PGLs [32,33]. Furthermore, not all of our patients have undergone genetic testing, as this has only been routinely carried out in our institution in recent years for smaller tympanic and tympanomastoid PGLs. To ensure the highest level of patient safety, it is advisable to follow the latest recommendations suggesting genetic testing for all patients, including isolated tympanic PGLs, until there is evidence that the risk of familial disease is negligible [4]. Given the high sensitivity of detecting *SDH* mutations using immunohistochemistry, it is advisable to immunohistochemically stain all samples collected for SDHB, especially if genetic testing is not available.

## 5. Conclusions

PGLs of stages A and B should be treated with surgery, which has been shown to be highly effective with a low complication rate. For patients with tumors growing to class C or higher, who declined surgery, or those who are unsuitable for major surgery, RT can be administered to halt tumor growth or even reduce its size. Long-term monitoring is advisable as recurrence can occur years after the initial treatment.

## Figures and Tables

**Table 1:** Management of PGLs according to tumor class (based on the modified Fisch classification).

Modified Fisch Classification of Temporal Bone PGLs	Number of Patients	Primary Treatment (Number of Patients)		
		Surgery	Radiation Therapy	Monitoring
A1	4	3		1
A2	5	4		1 #
B1	9	9 *		
B2	5	3		2 #

* Subsequent RT was administered in one patient, due to the residual tumor growth. # Monitoring was initially opted for due to surgery refusal, which was later followed by RT due to tumor growth.

**Table 2:** Organs at risk prioritization and acceptance criteria.

Structure	Priority Level	Parameter (Gy)	Constraints (Gy)	
			Desirable Dose	Acceptable Dose
Spinal cord	1	D_0.03 cm^3^_	≤45	≤50
Brain steam	1	D_0.03 cm^3^_	≤54	≤60
Optic chiasm	1	D_0.03 cm^3^_	≤54	≤60
Temporal lobe	2	D_0.03 cm^3^_	≤70	≤72
Optic nerve	3	D_0.03 cm^3^_	≤54	≤60
Eyeball	3	D_mean_/D_0.03 cm^3^_	D_mean_ ≤ 35	D_0.03 cm^3^_ ≤ 50
Lens	3	D_0.03 cm^3^_	≤6	≤15
Parotid gland	4	D_mean_	26	30 *
TM joint	4	D_2%_	≤70	≤75
Cochlea	4	D_mean_	≤45	≤55
Pituitary gland	4	D_0.03 cm^3^_	≤60	≤65
Mandible	4	D_2%_	≤70	≤75

* At least one gland. TM—temporomandibular; Dxx—the dose to the XX part of the structure volume.

## Data Availability

Data are contained within the article.
